# Acute ocular hypertension disrupts barrier integrity and pump function in rat corneal endothelial cells

**DOI:** 10.1038/s41598-017-07534-9

**Published:** 2017-07-31

**Authors:** Xian Li, Zhenhao Zhang, Lijun Ye, Jufeng Meng, Zhongyang Zhao, Zuguo Liu, Jiaoyue Hu

**Affiliations:** 1 Eye Institute of Xiamen University, Provincial Key Laboratory of Ophthalmology and Vision Science, Fujian, 361005 China; 2 Department of Ophthalmology, The Second Affiliated Hospital of the University of South China, Hunan, 421001 China; 3 Medical Technology Institute of Xuzhou Medical College, Jiangsu, 221004 China

## Abstract

Acute ocular hypertension (AOH) frequently compromises corneal endothelial cell (CEC) function in clinical practice. This type of stress induces corneal oedema and a decrease in the corneal endothelial cell density (ECD). The anterior chamber of the right eye of Sprague-Dawley rats was irrigated with Balanced Salt Solution (BSS) for two hours, and the left eye served as a control to determine the time-dependent effects of AOH on endothelial cell morphology and function. The average intraocular pressure (IOP) increased to 82.6 ± 2.3 mmHg (normal range: 10.2 ± 0.4 mmHg) during anterior irrigation. Very soon after initiating irrigation, corneal oedema became evident and the cornea exhibited a significant increase in permeability to FITC-dextran. The peripheral ECD was significantly reduced, and the morphology of CECs became irregular and multiform. The structures of the zonula occludens-1 (ZO-1) and F-actin were severely disrupted. In addtion, Na,K-ATPase exhibited a dispersed expression pattern. Two days after irrigation, obvious CEC proliferation was observed, the ECD recovered to a normal level, and F-actin was dispersed throughout the cytoplasm. Seven days later, the CEC structure and function were nearly normalized. Based on the results obtained using this model, an acute IOP crisis exerts transient deleterious effects on CEC structure and function in rats.

## Introduction

Glaucoma is the second leading cause of blindness worldwide, and the number of patients with glaucoma has increased^1^. Hypertensive glaucoma damages the optic nerve, causes a loss in the visual field, and simultaneously alters lens, iris and corneal endothelial cell (CEC) function^2^. CECs can adapt to a gradual and modest increase in intraocular pressure (IOP), even if it persists for an extended period, without exhibiting large changes^3^. In contrast, a rapid and transient increase in IOP induces corneal oedema. However, little is known about the mechanisms of this change in CECs. An acute, sudden, and large increase in IOP has been shown to influence the ultrastructural appearance of CECs^4, 5^ by disrupting the cytoplasm and causing pycnosis, excrescences and even a loss of CECs. This difference between the effects of a modest and gradual but prolonged increase and a sudden, large increase in IOP revealed that CECs were more vulnerable to and were damaged by an acute and large increase inIOP.

Acute primary angle closure (APAC) is one of the ophthalmic emergencies that is characterized by a sudden increase in IOP along with typical symptoms and clinical signs, such as corneal oedema, a shallow anterior chamber, blurred vision, severe ocular pain or headache, nausea and vomiting^6^. According to a large number of clinical studies, APAC leads to a significant decrease in endothelial cell density (ECD)^2, 7–12^ and increase in CEC pleomorphism and polymegathism^10^. Moreover, a decrease in ECD is negatively associated with the duration of the acute attack^2, 9, 10, 12^. In fact, corneal endothelial decompensation occurs when the ECD decreases to less than 400–700 cells/mm^2^ in patients. Corneal oedema is distinct in patients in the early stage of APAC and gradually disappears after the IOP decreases to a normal level. Obviously, the development of this type of corneal oedema is not caused by a decrease in ECD. The underlying pathogenic mechanisms that lead to early stage corneal oedema in patients with APAC are unclear.

The corneal endothelium is a monolayer covering the inner surface of the cornea. It provides a critical function in maintaining cornea transparency by regulating stromal deturgescence. Hydrophilic glycosaminoglycans constitute the stromal ground substance and are responsible for establishing a negative imbibition pressure that draws fluid into the interior of the stroma. Under steady-state conditions, the tendency of the stroma to swell is offset by endothelial osmotically driven fluid extrusion, which counter balances the stromal imbibition pressure. This dynamic balance required for maintaining corneal deturgescence is well known as the “pump-leak hypothesis” and is a prerequisite for cornea transparency^13^. Tight junctions (TJs) are an integral component of the corneal endothelial barrier. These junctional complexes include transmembrane proteins such as claudin and occludin, and membrane-associated proteins such as zonula occludens-1 (ZO)-1 and actin filaments^14^. ZO-1 plays an important role in maintaining the barrier function and serves as a TJ biomarker^15^. Moreover, Na,K-ATPase, which is located on the basolateral membrane of CECs, is primarily responsible for the pump function of the corneal endothelium and serves to transport Na^+^ ions coupled with bicarbonate from the corneal stroma to the aqueous humour^13, 16^. The barrier and “pump” functions of the corneal endothelium are responsible for maintaining corneal transparency^17, 18^. However, if this balance between the tendency to imbibe and export fluid is disrupted, stromal fluid accumulates and leads to corneal oedema. An increase in thickness of 20% or more is associated with the onset of translucence.

Even though a number of studies have reported an association between APAC and CEC function, researchers have not clearly determined whether APAC disrupts the CEC structure and function. By obtaining insights into this question, we will be able to determine whether an acute increase in IOP will disrupt the barrier integrity and “pump” function of CECs.

In this study, we used a rat model of acute ocular hypertension (AOH) to simulate the acute increase in IOP experienced by patients with APAC. Based on our results, an acute increase in IOP damages the structure and function of CECs, which is gradually reversed after the AOH has been resolved.

## Results

### Occurrence of corneal oedema

**Figure 1:**
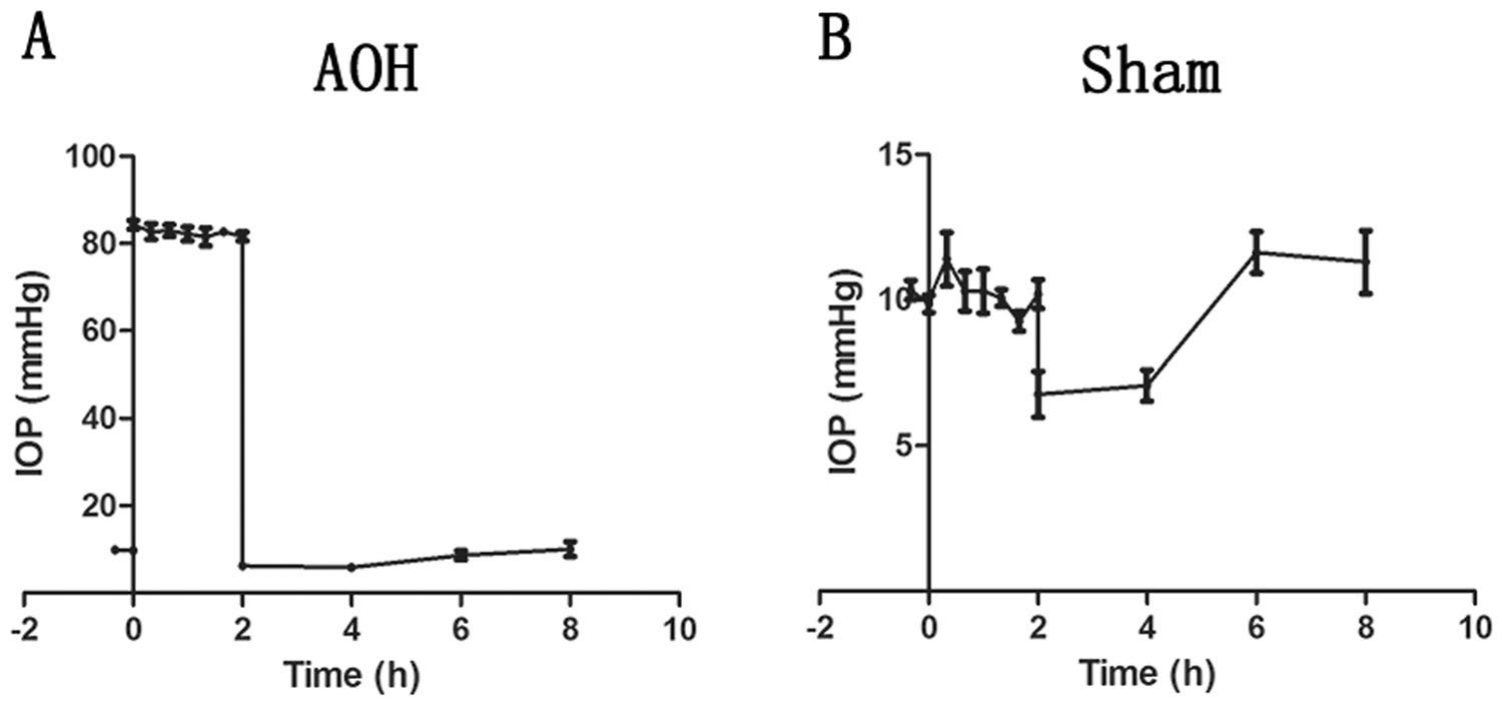
Changes in IOP observed in the AOH model and sham-operated rats. IOPs were evaluated in rats once every 20 minutes during irrigation and once every 2 hours after the irrigation using a hand-held, non-contact tonometer. The normal IOP of the rats was 10.2 ± 0.4 mmHg. At the beginning of irrigation, the IOP rapidly increased to 82.6 ± 2.3 mmHg (**A**). The IOP remained stable at 10.2 ± 1.0 mmHg in rats that underwent the sham procedure (**B**). After the infusion needle was removed, the IOP decreased to a level below the normal level and recovered to its baseline level after approximately 6 hours (**A** and **B**).

Initially, the normal IOP was 10.2 ± 0.4 mmHg and immediately increased to 82.6 ± 2.3 mmHg when the BSS container was elevated to 2.8 metres above the laboratory bench. In contrast, the IOP was sustained at 10.2 ± 1.0 mmHg in rats that underwent the sham procedure. After removing the infusion needle, the IOP decreased to a level slightly lower than the normal level, but returned to a normal level approximately 6 hours later (Fig. 1).

**Figure 2:**
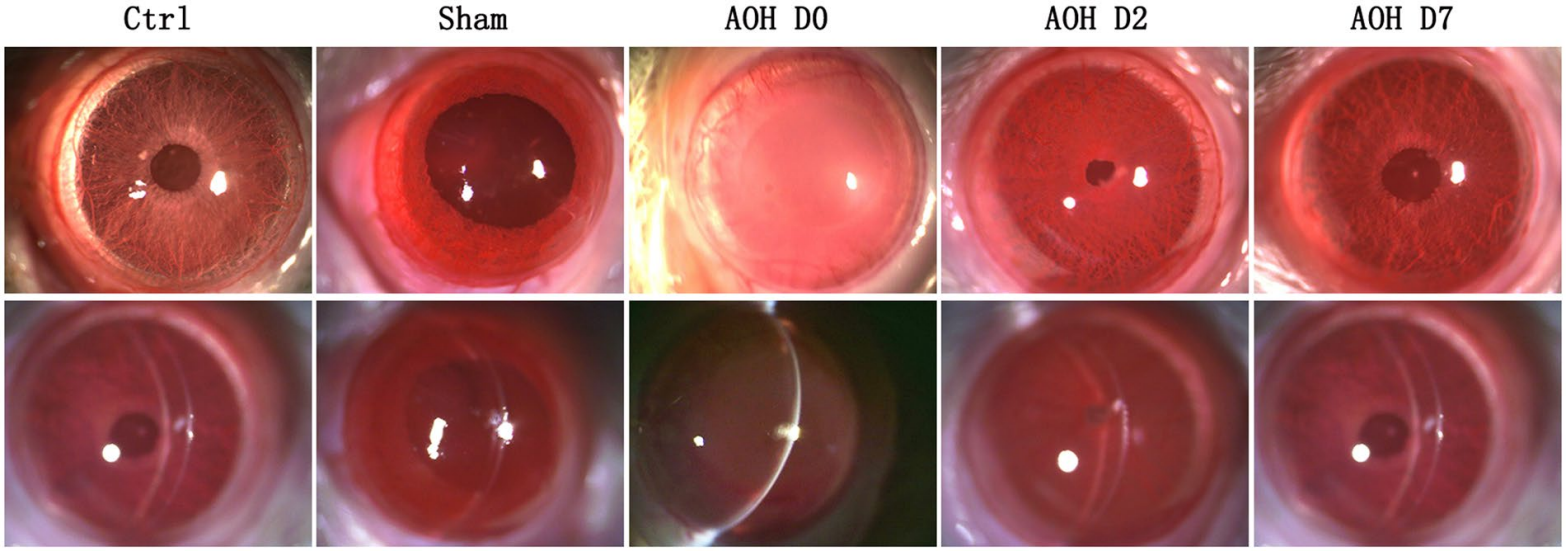
Slit lamp microscope examination of corneal images. The corneas of control and sham groups were transparent. Rapidly after irrigation started, the corneas became oedematous and opacified, and the iris borders were poorly defined. Two days later, corneal opacity gradually reversed to become transparent and the iris borders and structures were again well defined.

During the period in which the IOP was elevated, corneal oedema was induced within several minutes and the irrigated cornea became increasingly opaque, similar to the conditions observed in patients with APAC. Subsequent to the termination of the increased IOP, corneal transparency gradually increased and was essentially restored two days later. During this recovery phase, the normal appearance of the iris was also restored, as observed using a slit lamp microscopic examination. The corneas of the sham group always remained transparent in the absence of an increase in IOP (Fig. 2).

### Decreased peripheral corneal ECD

**Figure 3:**
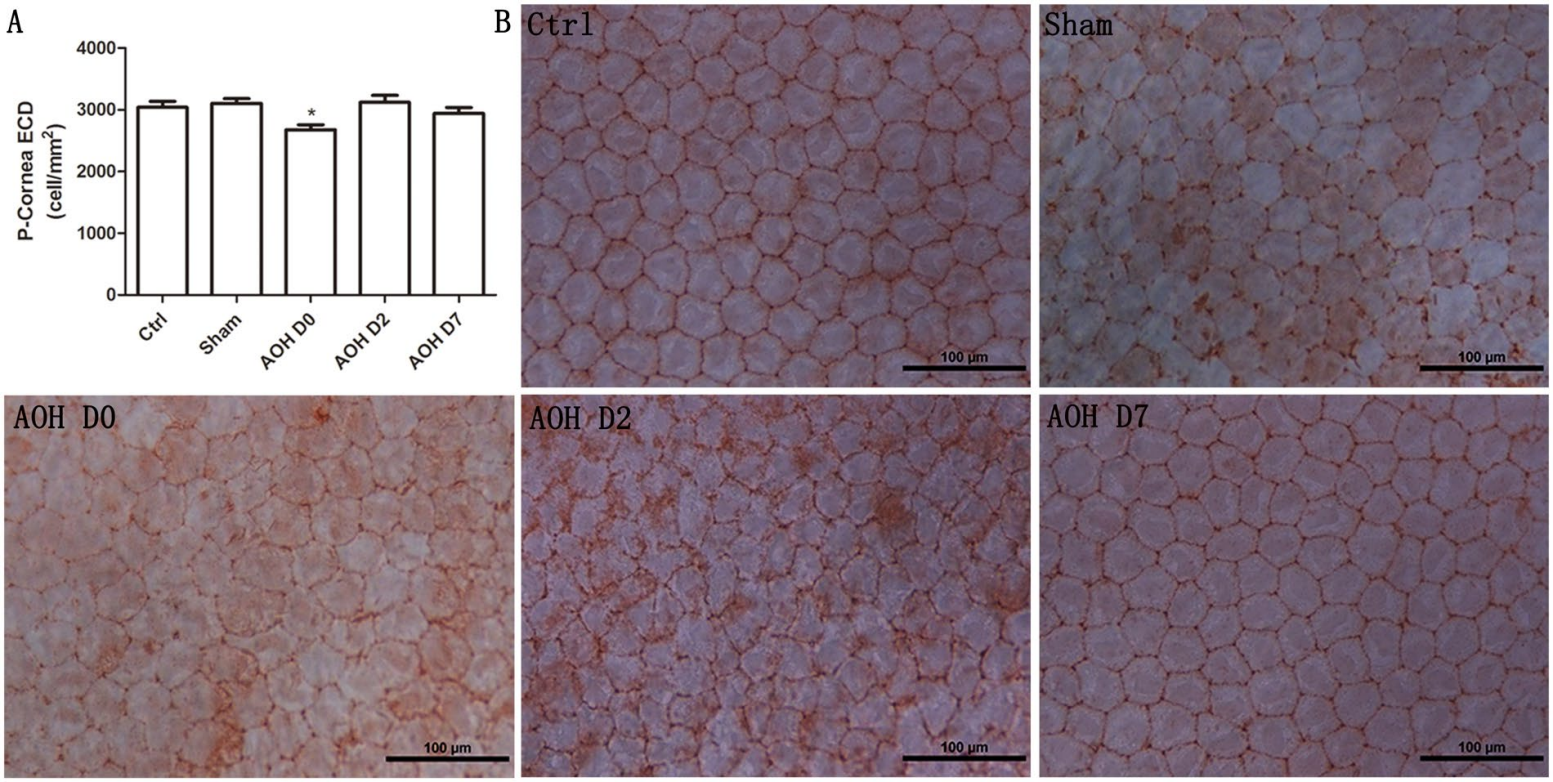
Peripheral CEC density quantification and histology. AOH decreased the peripheral corneal ECD (P < 0.05, Dunnett’s test) (**A**). Alizarin red staining revealed the CEC morphology (**B**). Normal CECs had a regular hexagonal morphology. Rapidly after irrigation, the morphology of CECs became irregular and multiform. Although the peripheral ECD increased to a normal level, the morphology was still not uniform after two days, whereas the morphology and size of the CECs had completely reversed to the normal condition after 7 days.

Alizarin red staining is a commonly used method to evaluate ECD and to visualize changes in CEC morphology. Similar to an earlier study^19^, the normal peripheral ECD was 3042 ± 308 cells/mm^2^. AOH resulted in a significant decrease in the peripheral ECD to 2678 ± 256 cells/mm^2^ (Fig. 3A). However, the ECD recovered to a normal level 2 days later. In contrast, the central corneal ECD remained unchanged (data not shown).

Normal CECs have a regular hexagonal morphology. Almost instantaneously after irrigation, the CEC morphology became irregular and multiform, and the volume of most cells increased. Two days later, the endothelial cells returned to their normal sizes, whereas their morphology was still diverse. Nevertheless, after 7 days, their size and morphology returned to normal (Fig. 3B).

### Proliferation of wounded CECs

**Figure 4:**
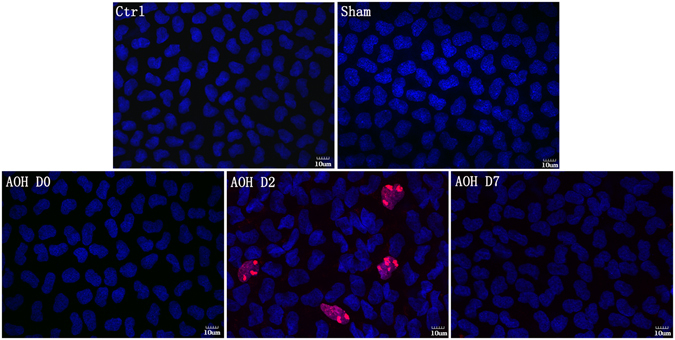
Changes in CECs induced by AOH. Ki67 immunostaining is used to examine cell proliferation. No Ki67-positive CECs were observed in controls. AOH rapidly decreased the peripheral ECD after irrigation. However, Ki67 staining was still negative. Two days later, Ki67-positive cells were readily apparent, indicating that AOH induced peripheral CECs to proliferate. At Day 7, Ki67-positive cells were not observed.

Rat CECs have a limited proliferative capacity^20, 21^. Ki67staining was not observed in control CECs. As mentioned above, the peripheral ECD was significantly decreased in rats that had been exposed to AOH for 2 hours. Nonetheless, Ki67 immunofluorescence (IF) staining was still negative. In contrast, two days later, Ki67-positive cells were observed (rate of positive: 5.2 ± 0.8%). However, no Ki67-positive cells were visible at Day 7 (Fig. 4).

### Disruption of the integrity of TJs

**Figure 5:**
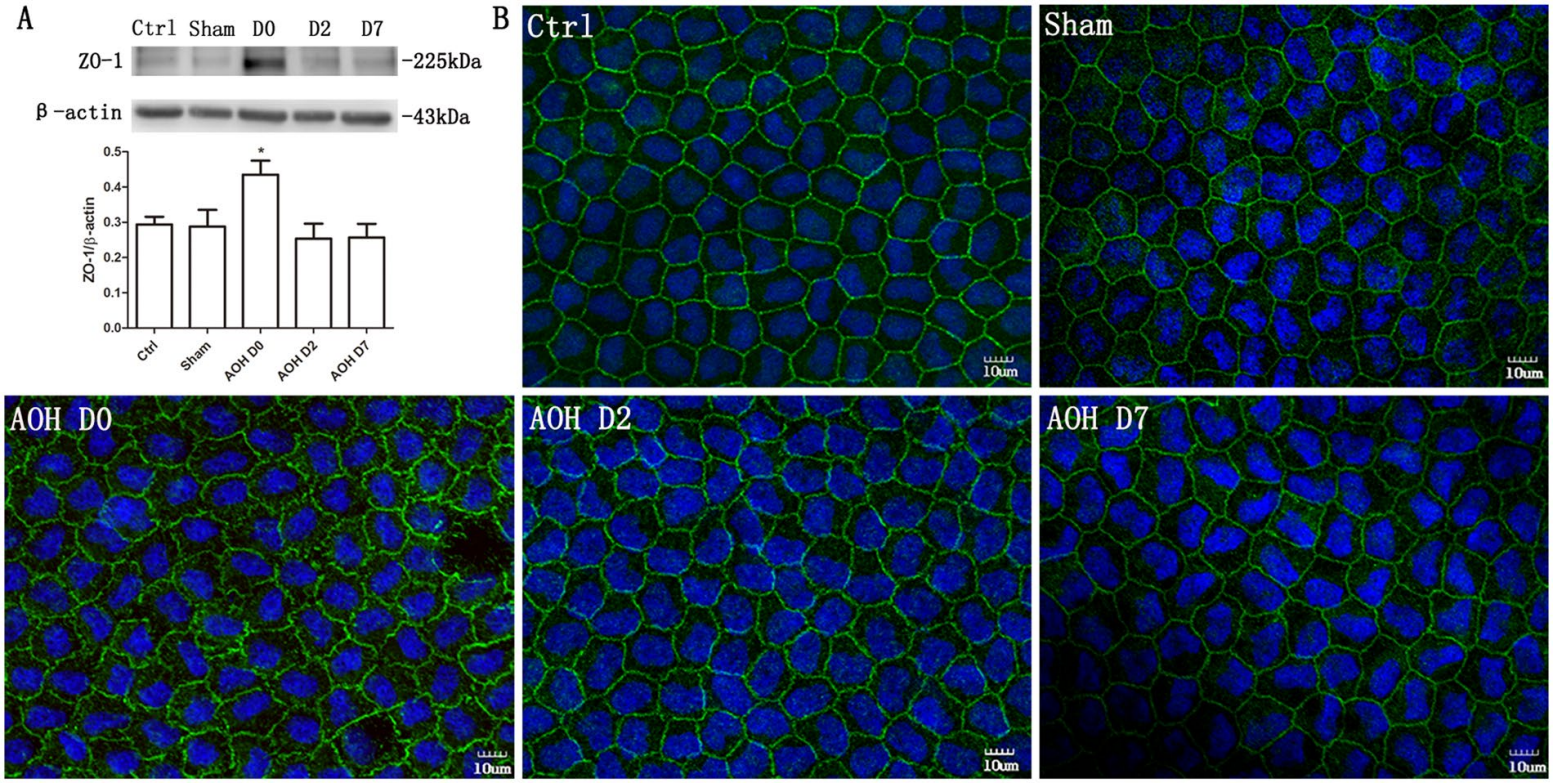
The integrity of the TJ protein ZO-1 was disrupted by AOH. AOH increased ZO-1 expression, based on a western blot analysis. The blots were run under the same experimental conditions and the images were from the same gel (**A**). Immunofluorescence staining for ZO-1 showed that ZO-1 formed a regular hexagonal pattern and was expressed in a contiguous pattern around the cell borders in the control group (**B**). The ZO-1 expression pattern was significantly disrupted by AOH, and ZO-1 became less localized to the cell borders and exhibited a discontinuous distribution. However, 2 days later, the normal ZO-1 distribution around the cell borders was restored.

ZO-1 is a TJ biomarker that plays an important role in maintaining the corneal endothelial barrier function. The distribution of ZO-1 was determined using immunofluorescence microscopy (Fig. 5B). In the corneal endothelium of the control group, ZO-1 formed a regular hexagonal pattern and was continuously expressed around the cell border. Almost instantaneously after irrigation, the ZO-1 expression patterns were significantly disrupted and ZO-1 expression in the cell borders became incomplete and discontinuous. However, 2 days later, the distribution of ZO-1 was mostly restored. The amount of ZO-1 in the corneal endothelium was confirmed by western blot analysis. Exposure to AOH for 2 hours induced a significant increase in ZO-1 expression (Fig. 5A).

### Cytoskeletal disorganization

**Figure 6:**
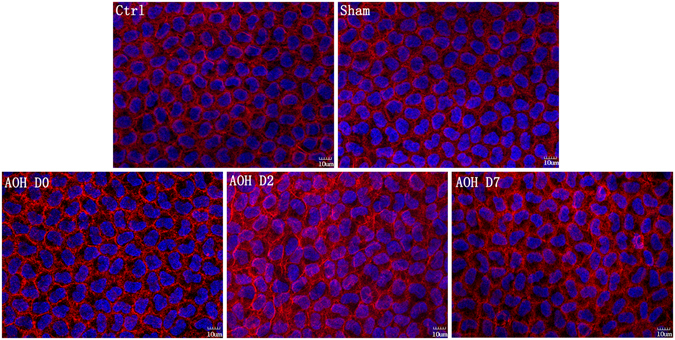
AOH disrupted the cytoskeletal (F-actin) organization. F-actin microfilaments were contiguous along the apical cell border and formed a circumferential, double-banded structure under control conditions. Rapidly after AOH, the double-banded structure was lost and F-actin exhibited a diffuse distribution. Two days later, the distribution of F-actin was not limited to the cell border but instead was diffusely scattered throughout the cytoplasm. The F-actin distribution only partially recovered and its double-banded structure reappeared in some cells at Day 7.

Texas Red-X phalloidin staining of the cytoskeleton (F-actin) was localized at the apical cell borders and produced a double-banded appearance in the normal corneal endothelium (Fig. 6). Almost instantaneously after AOH, the double-banded structure of endothelial cells disappeared and the F-actin expression pattern became diffuse. Furthermore, the F-actin distribution was no longer limited to the cell borders, but instead was scattered throughout the cytoplasm on Day 2. Seven days later, the F-actin distribution partially recovered and the cytoskeleton was partially reorganized.

### Disruption of Na, K ATPase localization

**Figure 7:**
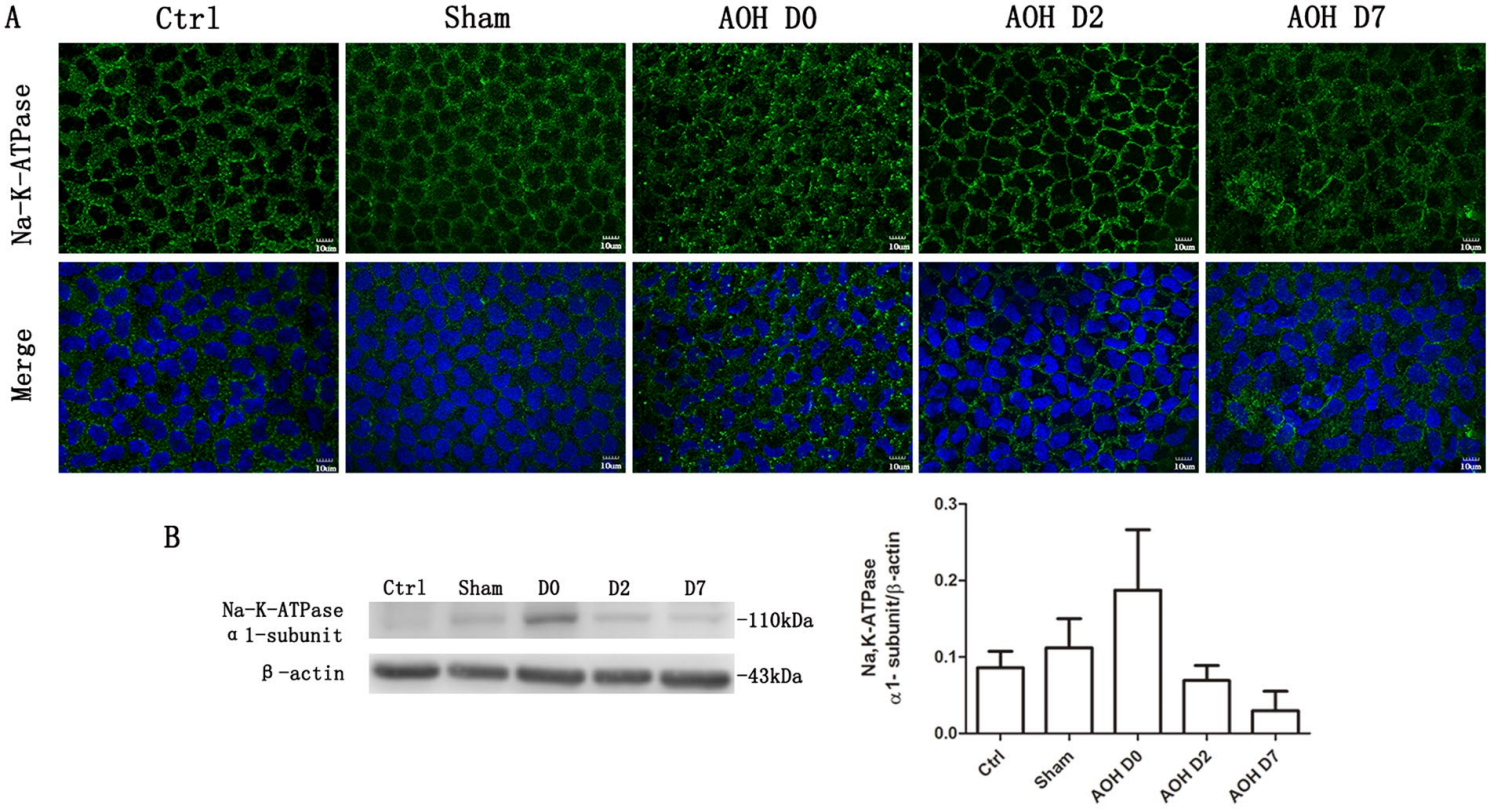
AOH disrupted the localization of Na,K-ATPase. Na,K-ATPase was localized to the basolateral membrane and was evenly distributed around the cell membrane in control corneas. AOH induced a dispersion of Na,K-ATPase away from its basolateral membrane localization. Two days later, the normal localization of Na,K-ATPase began to appear, but the recovery was still incomplete, even at Day 7 (**A**). AOH increased Na,K-ATPase expression very slightly, but not significantly, based on the western blot analysis. The blots were run under the same experimental conditions and the images were from the same gel (**B**).

Na,K-ATPase mediates net transendothelial ion transport and is delimited to the basolateral membrane in the corneal endothelium under control conditions. It is evenly and continuously expressed around the cell membrane. However, its localized expression became disrupted and scattered after the rats were exposed to AOH for 2 hours. Two days later, the Na,K-ATPase localization began to revert to its normal distribution. However, by Day 7, the recovery was still only partially complete (Fig. 7A). According to the results of the western blot analysis, the restoration of the Na,K-ATPase abundance was also incomplete at Day 7, and its level was not significantly different from the level observed at the end of the AOH procedure (Fig. 7B).

### Increase in corneal endothelial permeability

**Figure 8:**
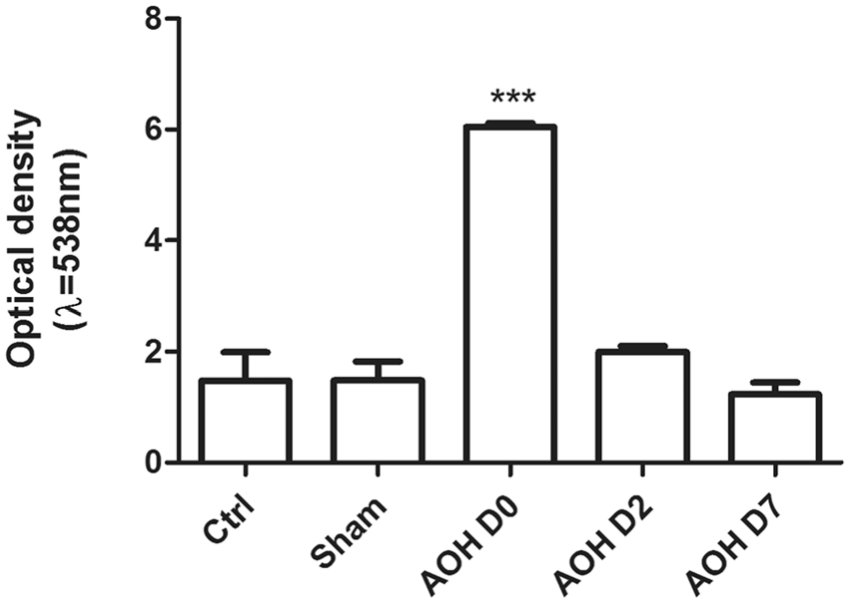
AOH induced an increase in transendothelial permeability. The influence of AOH on barrier integrity was evaluated by measuring FITC-dextran flux across the endothelium. The accumulation of FITC fluorescence in the entire cornea was detected using a microplate reader. AOH caused the permeability to increase. In contrast, two days later, FITC-dextran accumulation in the endothelial tissue decreased to normal levels. Data are presented as the means ± standard deviations (SD) from three independent experiments. ***P < 0.001 (Dunnett’s test).

The effect of AOH on corneal endothelium function was determined by measuring FITC-dextran accumulation in the tissue. The corneal endothelial barrier function was disrupted by exposure to AOH, since FITC-dextran permeability increased 4.1-fold compared with the control value measured immediately after termination of irrigation. Nevertheless, endothelial FITC-dextran accumulation decreased to a normal level, and the cornea again became transparent 2 days later (Fig. 8).

## Discussion

APAC is an ophthalmic emergency that damages the optic nerve, resulting in significant and permanent vision loss within several hours if it is not treated immediately^6^. Corneal oedema is one of the obvious clinical signs of this disease. We sought to obtain evidence to determine if the oedematous condition is associated with changes in CEC function and morphology. Our results revealed a good correlation between alterations in these attributes and AOH imposition. Almost immediately after irrigation, corneal oedema was obvious, as evidenced by a significant increase in FITC-dextran permeability in CECs. Decreased ECD in the peripheral cornea, barrier integrity, and CEC pump function induced corneal swelling and translucence. Nevertheless, two days later, corneal transparency was restored and Ki67-positive cells were observed, resulting in a restoration of the ECD. However, the CEC morphology remained irregular. Seven days after irrigation cessation, the CEC structure and function were nearly normalized, except for the expression patterns of F-actin and Na,K-ATPase.

AOH is a widely used model for inducing both retinal ischaemia and acute angle closure glaucoma. Melamed *et al*. identified vitreous irrigation as a procedure to minimize the damaging turbulence effects of the AOH perfusion operation on the endothelium^4^. In our procedure to induce AOH, we irrigated the anterior chamber, which has a somewhat larger volume than the volume of the vitreous cavity, using a previously described procedure^5^. Moreover, we included a sham procedure group to exclude any operation-induced artefacts. As the increased IOP was the main factor that damaged the endothelial cells^5^, the BSS irrigating solution was cautiously delivered to minimize any possible direct effects of turbulence. Corneal swelling rates greater than 33 μm/hr are associated with extensive compromise of endothelial cell integrity in some reports^22^. The swelling rate in this study was 24μm/hr, suggesting that our rate was less than the rate required to induce notable endothelial cell damage^23^.

In our protocol for inducing AOH, the peripheral ECD decreased. Because rat CECs exhibit limited proliferative capacity^21^ and Ki67-positive cells were detected on Day 2, ECD had recovered to a normal level. Although human CECs cannot proliferate *in vivo*, decreases in ECD are irreversible in patients afflicted with APAC^2, 7, 12^. Thus, in these individuals, the cornea is more susceptible to injury caused by surgical intervention. In clinical practice, the elevated IOP must be decreased within 2 hours, otherwise the ECD will irreversibly decrease and ultimately lead to CEC dysfunction.

We observed only a decrease in the peripheral CECs, consistent with the results from another study^5^; thus, in the AOH rat model, the increase in IOP is attributed to exogenous BSS irrigation. This procedure increased flow over the peripheral CEC surfaces. Nevertheless, further studies are required to clearly establish that shear stresses resulting from such flow accounted for the decreased ECD. In addition, tissue damage caused by the needle hole that was generated to provide access to the BSS perfusate may also have contributed to these effects. Furthermore, the needle hole was not completely sealed and thus some BSS exudation occurred. These conditions resulting from the AOH procedure are similar to the conditions encountered by patients who have undergone a trabeculectomy. These effects probably occur because an exudate that forms fluid blebs will exert increased pressure and possible CEC compression^24^. Therefore, we speculate that a change in the fluid dynamics of the aqueous humour contributes to peripheral CEC damage caused by our AOH procedure.

Three CEC-wound repair stages have been reported. In the first stage, adjacent endothelial cells migrate into the wound area and form temporary, incomplete tight junctions with minimal ion transporter pump sites. The second stage is characterized by barrier and pump function restoration. Simultaneously, the cornea regains its transparency and deturgescence, resulting in reversal of the oedematous condition, even though the endothelial cells still appear abnormal, with a polygonal shape rather than a cobblestone appearance. The third stage involves endothelial cell remodelling to restore their normal hexagonal shape^25^. Two days after BSS irrigation, the corneas became rather transparent. Despite the recovery of transparency, the results of the Alizarin red staining showed that the CECs still appeared pleomorphic and polymegathistic, suggesting that the wounded endothelial cells were in the second recovery stage^26^. Furthermore, the morphology of the endothelial cells regained a regular appearance at 7days, indicating that the rat CECs were nearly completely healed.

Generally, AOH disturbed ZO-1 and F-actin expression patterns, disrupted the distribution of Na,K-ATPase and induced a decrease in the ECD. These effects increased endothelial permeability as a consequence of losses in TJ barrier function, leading to corneal oedema. However, the underlying mechanisms precipitating these changes induced by an acute increase in IOP require further clarification.

## Materials and Methods

### Materials

Chloral hydrate was obtained from Abbott Laboratories (North Chicago, IL, USA). FITC-dextran (3–5 kDa), dimethyl sulfoxide (DMSO), Triton X-100 and paraformaldehyde were purchased from Sigma-Aldrich (St. Louis, MO, USA). Alizarin carmine was obtained from Chroma (Stuttgart, Germany). Texas Red-X phalloidin and the rabbit polyclonal antibody against ZO-1 were purchased from Zymed-Invitrogen (Carlsbad, CA, USA). Mouse monoclonal antibodies against Na,K- ATPase were obtained from Santa Cruz Biotechnology (Dallas, TX, USA), and the rabbit monoclonal antibody against Ki67 was purchased from Abcam (Cambridge, MA, USA). Alexa Fluor 488-conjugated donkey anti-rabbit IgG and Alexa Fluor 594-conjugated donkey anti-mouse IgG were purchased from Invitrogen-Gibco (Carlsbad, CA, USA). Hoechst 33342 and BSA were obtained from Vector Laboratories (Burlingame, CA, USA).

### Experimental animals

Sprague-Dawley (SD) rats, whose weight ranged from 250 to 300 g, were purchased from Shanghai Shilaike Laboratory Animal Co. Ltd. (Shanghai, China). Animals were housed in a temperature-and light-controlled room and had free access to food and water. All animals were cared for and treated in strict compliance with the Association for Research in Vision and Ophthalmology (ARVO) Statement for the Use of Animals in Ophthalmic and Vision Research. The protocols used in our study were approved by the Committee on the Ethics of Animal Experiments of Xiamen University (Permit Number: XMUMC2014-01-12).

### Rat model of AOH

An acute increase in IOP is a frequently used model of retinal ischaemia and may represent a model of APAC^27^. Eighty-four male SD rats were anaesthetized with intraperitoneal injections of 40 mg/kg pentobarbital. Pupillary dilation was maintained with 0.5% tropicamide (Santen Pharmaceutical Co.Ltd.), and corneal analgesia was achieved by a topical application of 0.5% oxybuprocaine (Alcon Laboratories). The anterior chamber of the right eye was cannulated with a 26-gauge infusion needle connected to a 500-ml reservoir of Balanced Salt Solution (BSS, Bausch & Lomb Incorporated). The IOP was evaluated with a hand-held, non-contact tonometer (HA-2, KOWA, Japan). The IOP increased to 82.6 ± 2.3 mmHg when the BSS container was elevated above the level of the anaesthetized rat for 2 hours. In patients afflicted with an APAC episode, the IOP has been reported to transiently increase to 64.8 ± 11.9 mmHg^11^, similar to the IOP observed in our rat model. A sham procedure was performed in which the infusion bottle was not elevated. The left eye also served as a control. At Days 0 (about several minutes), 2 and 7 after terminating the anterior chamber irrigation, all rats were euthanized and the corneas were rapidly excised.

### Slit lamp microscopic observation

The corneal oedema of each group was observed by three different observers under a slit lamp microscope (BQ900® Haag-Streit, Bern, Switzerland). The images were captured by an experienced researcher.

### Alizarin red staining

A solution of Alizarin red (0.1%) was diluted with 0.9% saline. The deep-iodine coloured solution was filtered (2-μm filter) to remove any undissolved sediment. The pH of the solution was then adjusted to 4.2 with diluted ammonium hydroxide (0.1% solution in normal saline). Excised corneas were placed in plastic vessels with the endothelial side up, immersed in an Alizarin red solution for 90 seconds, and then rinsed three times with saline to wash out the staining reagent. After the staining procedure, corneas were immersed in a 4% paraformaldehyde solution for 10 minutes at room temperature (RT) and then again rinsed three times with saline. Four radial incisions were made across each cornea to flat-mount it endothelial side up and examined under a Nikon Eclipse 50i light microscope (Nikon, Japan). The central cornea was regarded as the region in which the tissue diameter was less than 3mm, whereas the remaining regions were referred to as the peripheral cornea. Three images each were separately captured from the central and peripheral regions of the corneal endothelium in the 4 different quadrants. The ultimate ECDs for the central and peripheral areas were calculated by averaging the 12 ECDs.

### Immunofluorescence microscopy

The excised corneal tissues were fixed with 4% paraformaldehyde in PBS for 5 minutes at RT and in acetone for 3 minutes at −20 °C. After washing with PBS containing 1% Triton X-100 and 1% DMSO (TD buffer), the tissues were incubated in 2% BSA diluted in PBS for 1 hour at RT to block nonspecific binding and four radial incisions were made in each cornea. For F-actin staining, the corneas were incubated with Texas Red–X phalloidin (1:100) overnight at 4 °C. For ZO-1, Na,K-ATPase and Ki67 staining, tissues were incubated with primary antibodies against ZO-1 (1:100), Na,K-ATPase (1:100) or Ki67 (1:100) in a mixture of 1% BSA in PBS overnight at 4 °C. Subsequently, tissues were rinsed three times with TD buffer and incubated with secondary antibodies (Alexa Fluor 488/594-conjugated donkey anti-rabbit/mouse IgG, 1:200 in a mixture of 1% BSA in PBS) for 4 hours at RT, followed by three washes with TD buffer before the nuclei were counterstained with a 1:100 dilution of Hoechst 33342 dye. Stained whole-mount cornea tissues were mounted endothelial side up on a slide using H-1000. Finally, the corneal tissues were visualized under a laser confocal microscope (Olympus Fluoview 1000; Olympus).

### Permeability of the corneal endothelium to FITC-dextran

The permeability of the corneal endothelium was evaluated by measuring FITC-dextran accumulation in the corneal tissue^28, 29^. Rats were anaesthetized with 10% chloral hydrate to establish the AOH model. Ten microliters of FITC-dextran (3–5 kDa) in PBS (1 mg/ml) were injected into the anterior chamber through a micro-injector. After 10 minutes, the corneas were excised, quickly rinsed three times with PBS, and then homogenized in 300 μl of PBS. Subsequently, the medium was centrifuged at 9,000 × *g* for 10 minutes at 4 °C. The supernatants were collected to measure FITC fluorescence using a SpectraMax M2e Microplate Reader (Molecular Devices, Sunnyvale, CA), with an excitation wavelength of 485 nm and an emission wavelength of 538 nm.

### Western blot analysis

CECs were removed from each group using Algerbrush II and then the remaining corneal tissues were immersed into cell lysis buffer for 2 hours without being cut into fragments to detect the expression of ZO-1 and Na, K-ATPase in the corneal endothelium. Cell lysates containing equal amounts of protein were subjected to electrophoresison 8% SDS-PAGE gels (ZO-1, 225KDa; Na,K-ATPase, 110 KDa) and then electrophoretically transferred onto a PVDF membrane. After blocking with 2% BSA for 1 hour at RT, the membranes were incubated with primary antibodies against ZO-1 (1:500), Na,K-ATPase (1:250) or the loading control β-actin (1:10,000) overnight at 4 °C with gentle rocking. After three washes with Tris-buffered saline containing 0.05% Tween-20 for 10 minutes each, the membranes were incubated with horseradish peroxidase (HRP)-conjugated goat anti-rabbit/mouse IgG (1:10,000) for 1 hour at RT. The blots were visualized using an enhanced chemiluminescence (ECL) reagent. Band intensities were measured using a Molecular Imager ChemiDocXRS System (Bio-Rad, Hercules, CA) and analysed with image analysis software (Quantity One; Bio-Rad).

### Statistical analysis

Quantitative data are presented as the means ± standard errors (SE) from three independent experiments. Differences were evaluated using ANOVA followed by Dunnett’s multiple comparison tests. A P value of less than 0.05 was considered statistically significant.

### Data availability

All data generated or analysed during this study are included in this published article.

## Electronic supplementary material

Supplementary information
