# Supplementary material for: Acute ocular hypertension disrupts barrier integrity and pump function in rat corneal endothelial cells
Source: Sci Rep. 2017 Jul 31;7:6951. doi: 10.1038/s41598-017-07534-9 (PMC5537405; doi:10.1038/s41598-017-07534-9)
Supplement: Supplementary file 1 — Supplementary information [file 41598_2017_7534_MOESM1_ESM.pdf]

# Acute ocular hypertension disrupts barrier integrity and pump function in rat corneal endothelial cells

**Xian Li[1,2, #], Zhenhao Zhang[1,3, #], Lijun Ye[1], Jufeng Meng[1], Zhongyang Zhao[1], Zuguo Liu[1, *], and Jiaoyue Hu[1, *]**

1. Eye Institute of Xiamen University, Provincial Key Laboratory of Ophthalmology and Vision Science, Fujian, 361005, China

2. Department of Ophthalmology, The Second Affiliated Hospital of the University of South China, Hunan, 421001, China

3. Medical Technology Institute of Xuzhou Medical College, Jiangsu, 221004, China

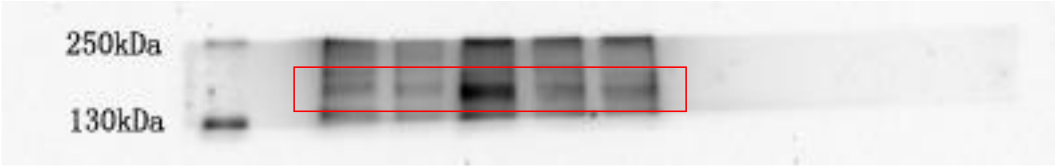


Figure S1 Representative images of western blot of ZO-1 in the corneal endothelial cells of rat. The blots were run under the same experimental conditions and the images were from the same gel.

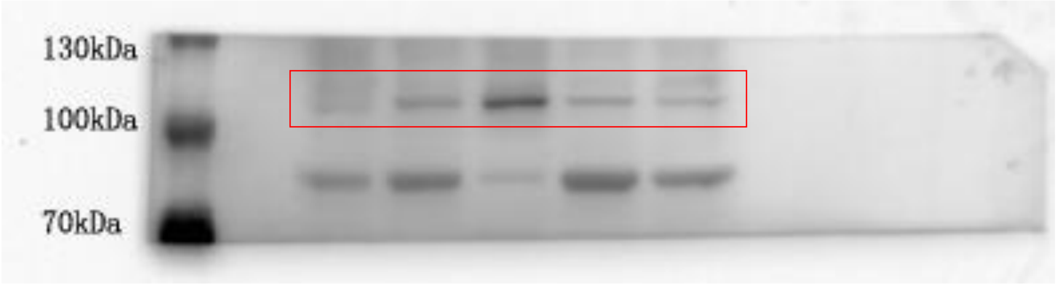


Figure S2 Representative images of western blot of Na,K-ATPase in the corneal endothelial cells of rat. The blots were run under the same experimental conditions and the images were from the same gel.
